# Supplementary material for: Thrombopoietin increases susceptibility for EVI1 + KMT2A-MLLT3-driven AML expressing stem cell genes linked to poor outcome
Source: Nat Commun. 2025 Dec 19;17:892. doi: 10.1038/s41467-025-67611-w (PMC12830621; doi:10.1038/s41467-025-67611-w)
Supplement: Supplementary file 1 — Supplementary Information [file 41467_2025_67611_MOESM1_ESM.pdf]

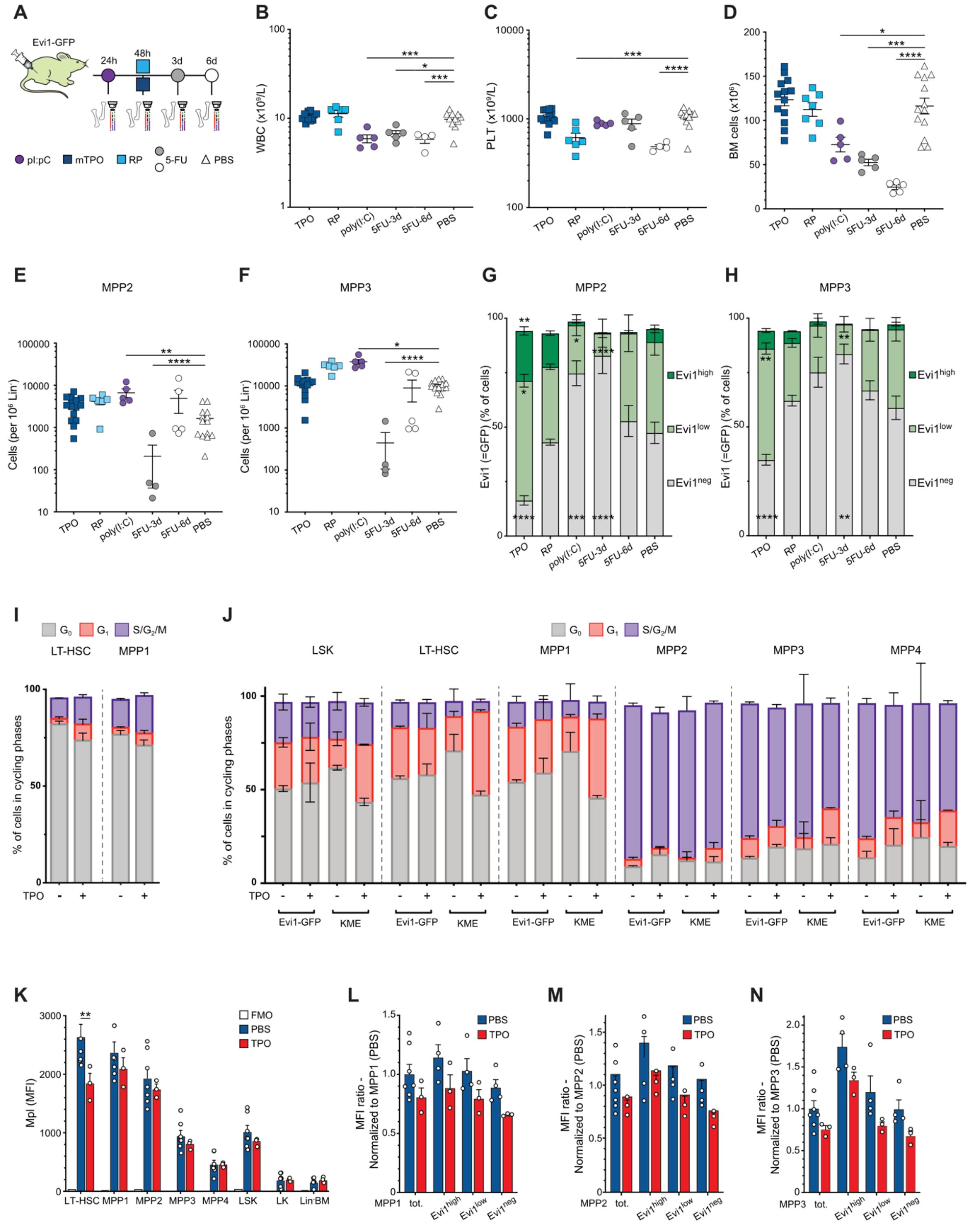

Supplementary Figure 1

TPO exposure increases cycling of Evi1^{high} LT-HSC and MPP1 cell fractions

(A) Experimental setup: Evi1$^{GFP/+}$ mice were injected with poly(I:C), TPO, RP and 5-FU, and then BM cells were analysed after 24h (poly(I:C)), 48h (TPO, RP), or 3 and 6 days (5-FU) respectively. **(B-D)** Peripheral WBC and PLT counts, as well as BM cellularity of mice treated with TPO (n=13), RP (n=6 except for D: n=7), pI:pC (n=5), 5FU-3d (n=5), 5FU-6d (n=4 except for D: n=5) or PBS (n=11 except for D: n=13). **(E-F)** Number of MPP2 and MPP3 cells (normalized to 10^6 Lin$^-$BM cells) in KME mice treated with the different factors (TPO n=15, RP n=6, poly(I:C) n=5, 5FU-3d n=4, 5FU-6d=5, PBS n=14). **(G-H)** Flow cytometric quantification

of the proportion (%) of MPP2 **(F)** and MPP3 **(G)** expressing $Evi1^{high}$, $Evi1^{low}$ and $Evi1^{neg}$ following the different treatments. (TPO: n=15, RP: n=6, pI:pC: n=5, 5-FU 3d: n=4, 5-FU 6d: n=5, PBS: n=13). **(I)** Flow cytometric quantification of the proportion of LT-HSC and MPP1 in the different phases of the cell cycle 48h after PBS (n=2) or TPO (n=3) treatment. Steady-state LT-HSC: 81.9%±1.6; G1: 2.9%±0.99; S/G2/M: 10.5%±0.25. **(J)** Flow cytometric quantification of the proportion (%) of LSK, LT-HSC, MPP1-4 in Evi1-GFP and in iKMT2A-MLLT3 mice (off-DOX) after exposure to TPO (or PBS control). **(K)** Flow cytometric quantification of Mpl expression (MFI) on different HSPC populations from mice 48h after treatment with TPO (n=3) or PBS (n=7). **(L-N)** Mpl expression (MFI) in MPP1, MPP2 and MPP3 cells expressing different levels of Evi1 (high, low or negative) from TPO-treated KME mice (n=3) normalized to Mpl (MFI) of total MPP1, MPP2 or MPP3 respectively from the PBS-treated controls (n=7). n = number of individual mice analyzed. Statistically significant differences to PBS controls are shown in the plots. 1-way ANOVA **(B-F)** and 2-way ANOVA **(G-N)** followed by two-sided unpaired t-test (**B-F, I&J**), Tukey's post-test (**G&H**) or Sidak multiple comparison test (**K-N**) used to determine significance and data are represented as mean±SEM (*: p<0.05; **: p<0.01; ***: p<0.001; ****: p<0.0001). For bar- and dot-plot figures, source data are provided in the Source Data file.

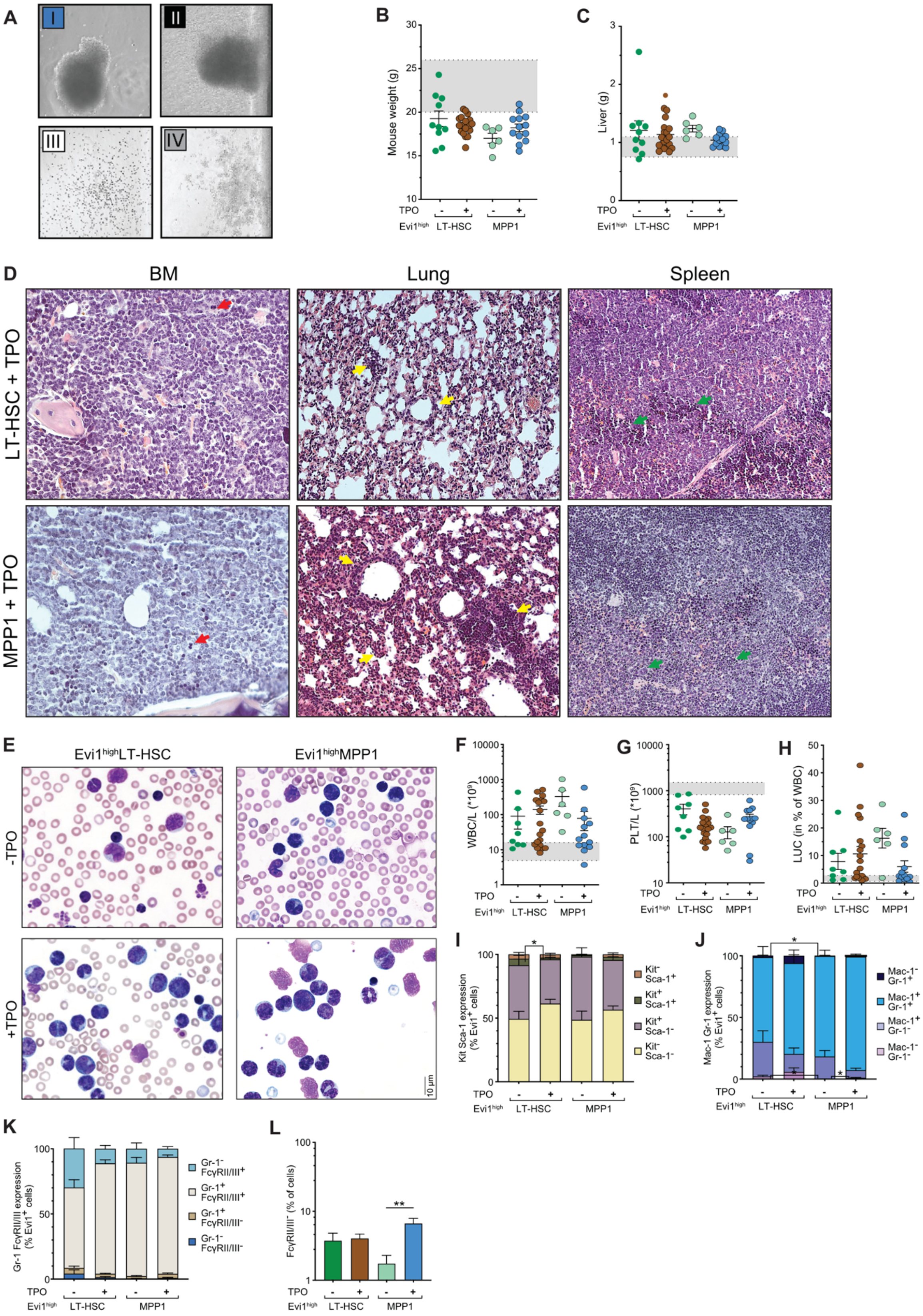

Supplementary Figure 2

TPO increases colony formation and accelerates iKMT2A-MLLT3-driven AML

(A) Types of colonies formed by iKMT2A-MLLT3 murine AML cells in growth factor-containing MC. Type I colonies are dense with sharp edges; Type II are dense with loose edges, Type III show no clear core, and Type IV colonies have ill-defined cores with multiple extensions. The latter are typically formed by HSC-derived AML cells[9]. Body- **(B)** and liver weight **(C)** from diseased mice transplanted with Evi1^{high} iKMT2A-MLLT3 LT-HSC (+TPO n=20, PBS n=10) or -MPP1 (+TPO n=13, PBS n=6) with and without pre-treatment. Mice transplanted with LT-HSC derived cells are shown in dark green & brown dots, while mice transplanted with MPP1-derived cells are shown in light green and blue dots (also in panels F-H). Grey areas represent the average body- and liver weight in healthy mice. **(D)** Representative HE-stained sections of BM, lung, and spleen from diseased mice upon transplantation with LT-HSC (top) or MPP1 (bottom) from TPO-pre-treated KME donors. Scale bar = 20μm; 40x magnification. Arrows indicate leukemic blasts. **(E)** Representative images of Wright-Giemsa-stained peripheral blood smears from symptomatic mice at sacrifice. Scale bar = 10μm; 60x magnification. **(F-H)** WBC**,** PLT, and large unidentified cells (LUC≈blasts) counts in the peripheral blood of symptomatic transplanted mice LT-HSC (+TPO n=19, PBS n=8) or -MPP1 (+TPO n=13, PBS n=6) at sacrifice. **(I-K)** Flow cytometric characterization of the Evi1^{+} cells from diseased mice transplanted with LT-HSC (+TPO n=19, PBS n=8) or MPP1 (+TPO n=13, PBS n=6). Expression of and Kit/Sca-1, Mac-1/Gr-1, and Gr-1/FcγRII/III. **(L)** Proportion (%) of FcγRII/III^{-} (CD16/32) BM cells from diseased mice (same as Fig. I-K) transplanted with LT-HSC or MPP1 from TPO-treated or controls. n = number of individual mice analyzed. Statistical significance was calculated with 1-way ANOVA **(B&C, F-H, L)** and 2-way ANOVA **(I-K)** followed by Tukey's post-hoc were used to test for significance and data are represented as mean±SEM (*: p<0.05; **: p<0.01; ***: p<0.001; ****: p<0.0001). For bar- and dot-plot figures, source data are provided in the Source Data file.

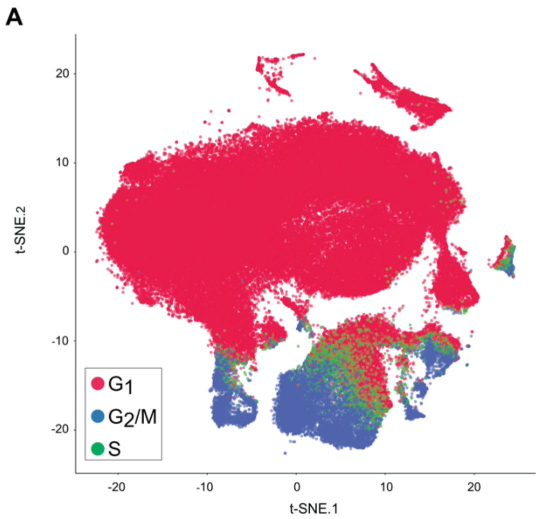

<u>**Supplementary Figure 3**</u>

Analysis of cell cycle phases

(A) t-SNE dimensionality reduction plot, with colour illustrating the cycling phase-related cell annotations obtained by the Cyclone prediction tool[93]

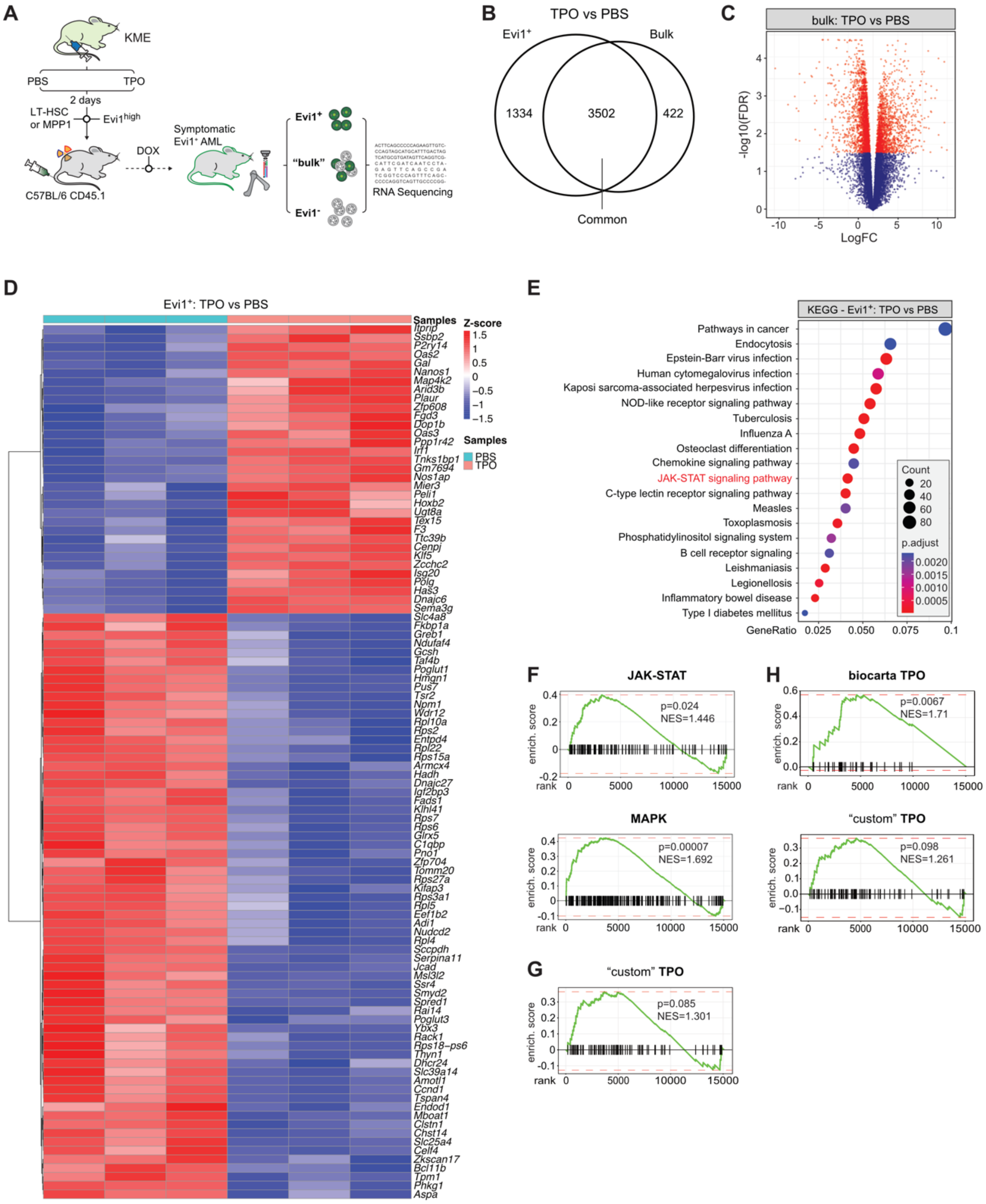

Supplementary Figure 4

Bulk RNAseq analysis of TPO-treated KME HSPC

(A) Experimental setup: wildtype mice were transplanted with KME HSPC (LT-HSC or MPP1) from donors pre-treated for 48h with a single dose of TPO or PBS. The gene expression signatures from emerging AML cells either expressing Evi1 (GFP^+) or not (GFP^-), as well as the mixed population ("bulk") were compared. **(B)** Venn diagram showing the overlap of DEGs of KME AML cells emerging from TPO or PBS treated mice between the different fractions ($Evi1^+$ vs bulk). **(C)** Volcano plot showing DEGs in the bulk fraction of AML cells emerging from TPO- or PBS-treated mice. **(D)** Heatmap of the DEGs in the $Evi1^+$ fraction of AML cells emerging from TPO- vs PBS-treated mice. **(E)** Analysis of common DEG revealed up-regulation of KEGG pathways linked to JAK-STAT and NF-κB signalling pathways. **(F)** GSEA

revealed significant upregulation of JAK-STAT (top) and MAPK (bottom) pathways related genes in Evi1$^+$ blasts from symptomatic mice transplanted with TPO-stimulated KME BM cells. **(G)** GSEA revealed significant upregulation of TPO pathway[44] related genes in bulk blasts from symptomatic mice transplanted with TPO-stimulated cells. **(H)** GSEA revealed significant upregulation of custom TPO[44] and Biocarta (Msig-DB) TPO pathway-related genes in Evi1$^+$ blasts from symptomatic mice transplanted with TPO-stimulated cells. p-values were calculated with quasi-likelihood F-tests from EdgeR package **(C)** or permutation test of GSEA function from clusterProfiler R package **(E-G)**.

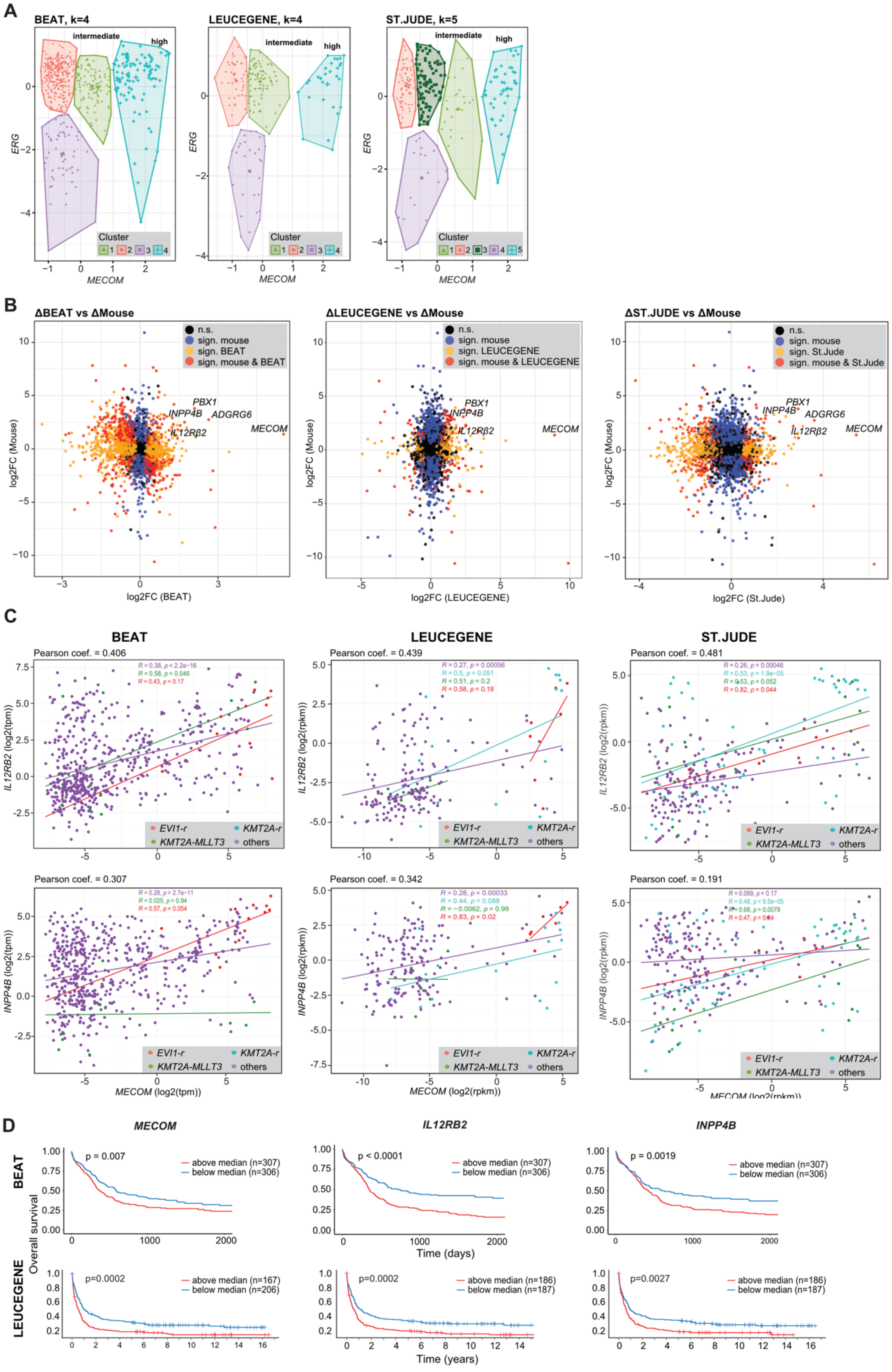
A
BEAT, k=4
LEUCEGENE, k=4
ST.JUDE, k=5
intermediate
high
ERG
MECOM
Cluster
B
ΔBEAT vs ΔMouse
ΔLEUCEGENE vs ΔMouse
ΔST.JUDE vs ΔMouse
n.s.
sign. mouse
sign. BEAT
sign. mouse & BEAT
sign. LEUCEGENE
sign. mouse & LEUCEGENE
sign. St.Jude
sign. mouse & St.Jude
PBX1
INPP4B
ADGRG6
IL12Rβ2
MECOM
log2FC (Mouse)
log2FC (BEAT)
log2FC (LEUCEGENE)
log2FC (St.Jude)
C
BEAT
LEUCEGENE
ST.JUDE
Pearson coef. = 0.406
Pearson coef. = 0.439
Pearson coef. = 0.481
Pearson coef. = 0.307
Pearson coef. = 0.342
Pearson coef. = 0.191
IL12RB2 (log2(tpm))
IL12RB2 (log2(rpkm))
INPP4B (log2(tpm))
INPP4B (log2(rpkm))
EVI1-r
KMT2A-r
KMT2A-MLLT3
others
MECOM (log2(tpm))
MECOM (log2(rpkm))
D
MECOM
IL12RB2
INPP4B
BEAT
LEUCEGENE
Overall survival
p = 0.007
p < 0.0001
p = 0.0019
p=0.0002
p=0.0002
p=0.0027
above median (n=307)
below median (n=306)
above median (n=167)
below median (n=206)
above median (n=186)
below median (n=187)
Time (days)
Time (years)

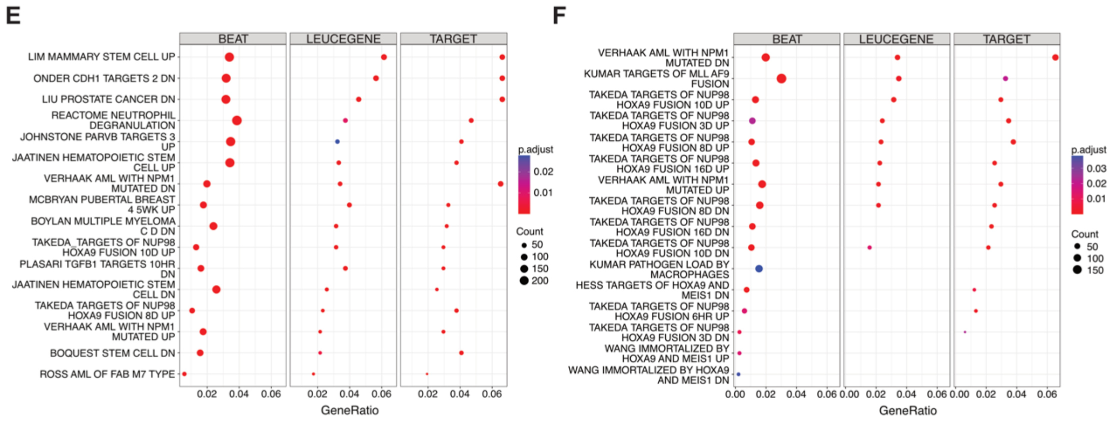

Supplementary Figure 5

Comparison of expression signatures from TPO-stimulated HSC-derived murine Evi1^{+}AML with human EVI1^{+}AML reveals common HSC genes associated with poor outcome

(A) k-means clustering of AML patients from the BEAT (left), LEUCEGENE (right) and ST.JUDE (right) cohorts according to ERG and MECOM expression. **(B)** Scatterplots comparing DEG from the Evi1^{+} (TPO vs PBS-treated origin) mouse AML signature and the DEG in EVI1^{high} vs EVI1^{intermediate} patients from the BEAT (left), LEUCEGENE (middle) and ST.JUDE (right) cohorts. **(C)** Scatterplots showing co-expression of the highest up-regulated genes IL12Rβ2 (top) and INPP4B (bottom) vs MECOM in the patients of the BEAT (left), LEUCEGENE (middle) and ST.JUDE (right) AML cohorts. Pearson correlations were calculated for all patients (on the top), or only in group of patients with Evi1-r, KMT2A-r, in KMT2A-MLLT3 or without KMT2A genomic rearrangements (coloured in red, blue, green and purple respectively). Two-sided Pearson correlation test was used to calculate the significance for each group. **(D)** Survival curves for the BEAT (top) and LEUCEGENE (bottom) patients split according to the median gene expression of MECOM (left), IL12Rβ2 (middle) and INPP4B (right). p-values were calculated with Log-rank test. **(E)** ORA analysis revealed associations to signatures (Msig C2) of “Haematopoetic stem cells” and aberrant Hoxa9 activity in the Evi1^{high} patients (MSigD pathways). p-values were calculated by a hypergeometric distribution with enricher function from clusterProfiler R package. **(F)** ORA analysis revealed enrichment of signatures of “Hoxa9-MLL/ENL”, “Targets of MLL-AF9” and “NUP98-Hoxa9 fusion” (MSigD pathways). p-values were calculated by a hypergeometric distribution with enricher function from clusterProfiler R package.

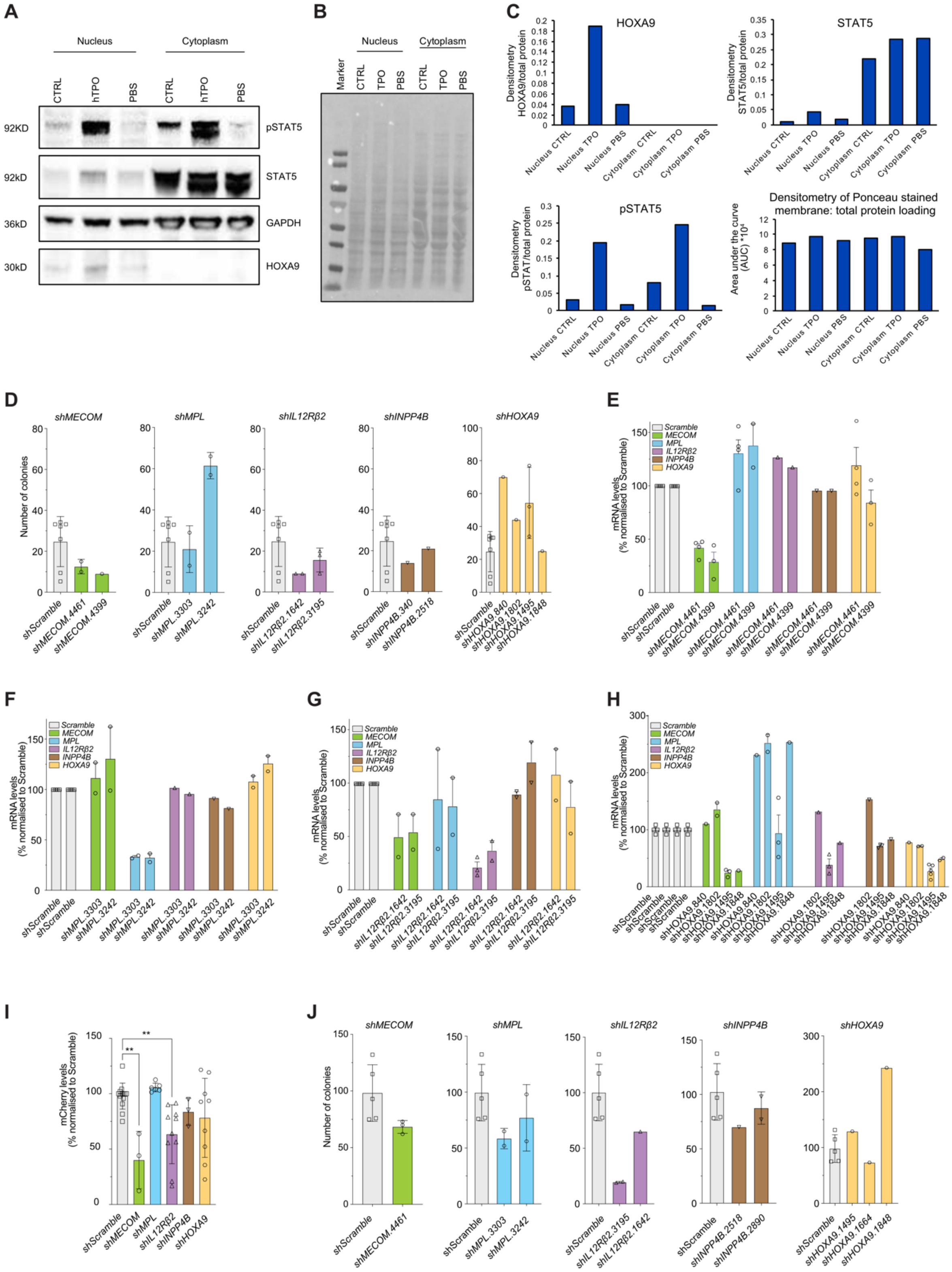

A
Nucleus
Cytoplasm
CTRL
hTPO
PBS
92KD
pSTAT5
92kD
STAT5
36kD
GAPDH
30kD
HOXA9
B
Marker
CTRL
TPO
PBS
C
HOXA9
Densitometry HOXA9/total protein
STAT5
Densitometry STAT5/total protein
pSTAT5
Densitometry pSTAT/total protein
Densitometry of Ponceau stained membrane: total protein loading
Area under the curve (AUC) *10⁴
Nucleus CTRL
Nucleus TPO
Nucleus PBS
Cytoplasm CTRL
Cytoplasm TPO
Cytoplasm PBS
D
shMECOM
shMPL
shIL12Rβ2
shINPP4B
shHOXA9
Number of colonies
shScramble
shMECOM.4461
shMECOM.4399
shMPL.3303
shMPL.3242
shIL12Rβ2.1642
shIL12Rβ2.3195
shINPP4B.340
shINPP4B.2518
shHOXA9.840
shHOXA9.1802
shHOXA9.1495
shHOXA9.1848
E
Scramble
MECOM
MPL
IL12Rβ2
INPP4B
HOXA9
mRNA levels (% normalised to Scramble)
F
G
H
I
mCherry levels (% normalised to Scramble)
**
shMECOM
shMPL
shIL12Rβ2
shINPP4B
shHOXA9
J
shINPP4B.2890
shHOXA9.1664

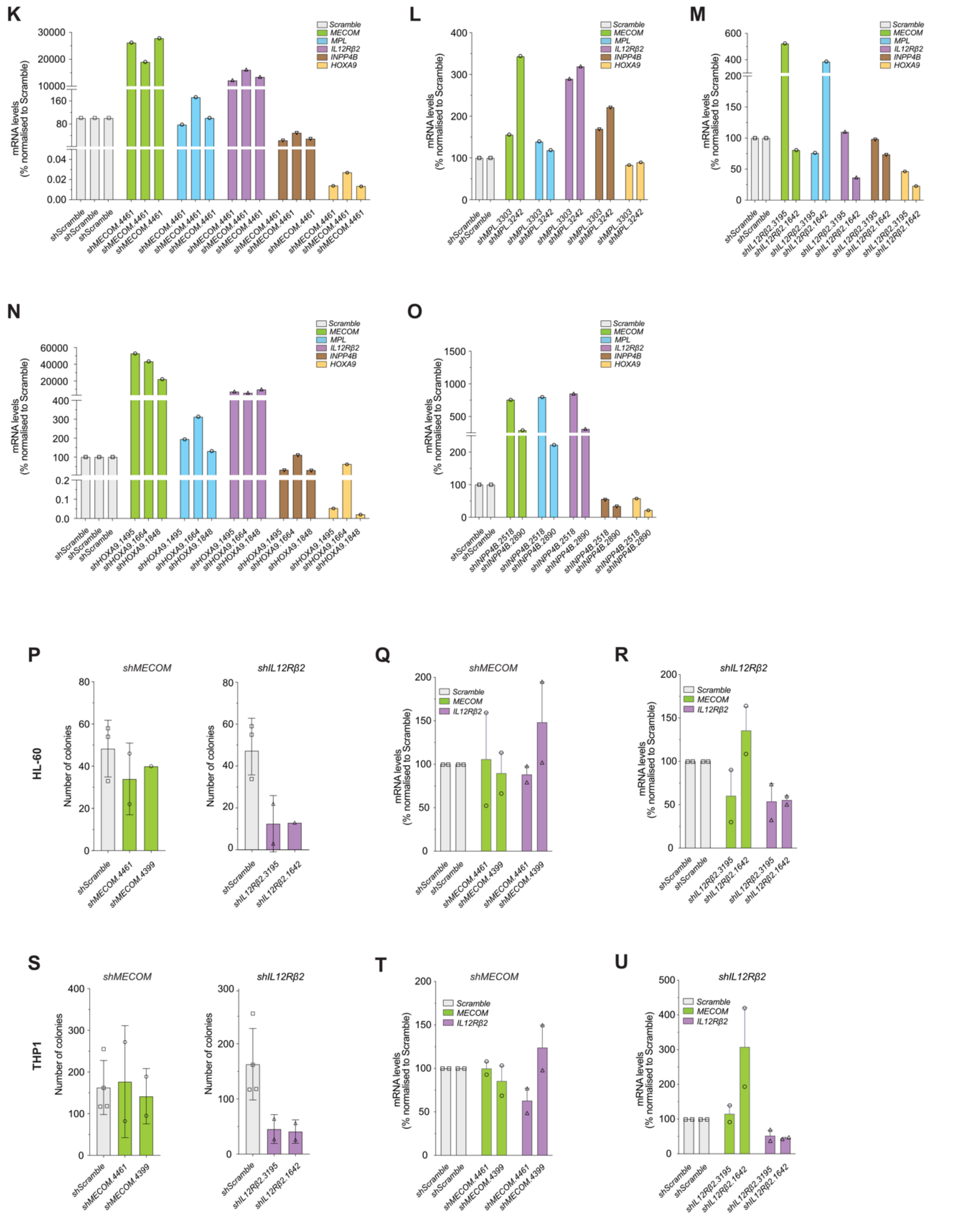

Supplementary Figure 6

Functional validation of top-upregulated DEG in human and mouse KMT2A-r EVI1⁺AML cells

(A) Western Blot of nuclear and cytoplasmic fractions of OCI-AML4 cells starved for 4h followed by short-term TPO stimulation showed increased phosphorylation of STAT5, accompanied with an increase of nuclear HOXA9. **(B)** Ponceau-stained Western blot membrane, showing equal loading in all lanes, was used for densitometric quantification and total protein normalization. **(C)** Densitometric quantification of the observed bands in nuclear and cytoplasmic fractions for HOXA9, STAT5, and pSTAT5. AUC (area under the curve) values

of the bands were normalized using total protein AUC values. **(D)** Number of colonies formed by OCI-AML4 cells expressing the indicated shRNA targeting MECOM, MPL, IL12Rβ2, HOXA9 or INPP4B compared to the Scramble controls after 14 days in MC (H4534). **(E-H)** RT-qPCR-assessed mRNA levels of MECOM, MPL, IL12Rβ2, INPP4B and HOXA9 in OCI-AML4 virally expressing shRNA targeting MECOM **(E)**, MPL **(F)**, IL12Rβ2 (**G**) or HOXA9 **(H)**. **(I)** Percentage of mCherry-positive OCI-AML4 cells expressing MECOM, MPL, IL12RB2, INPP4B, HOXA9 or Scramble shRNA, harvested from MC plates**.** Dunnett's multiple comparisons test was used to test for significance. **(J)** Number of colonies formed by MOLM-13 cells expressing the indicated shRNA targeting MECOM, MPL, IL12Rβ2**,** HOXA9 or INPP4B compared to the Scramble controls after 14 days in MC (H4534). **(K-O)** RT-qPCR-assessed mRNA levels of MECOM, MPL, IL12Rβ2, INPP4B and HOXA9 in MOLM-13 cells virally expressing shRNA targeting MECOM **(K)**, MPL **(L)**, IL12Rβ2 (**M**), HOXA9 **(N)** or INPP4B **(O)**. **(P)** Number of colonies formed by HL-60 cells expressing shRNA targeting MECOM or IL12Rβ2 compared to the Scramble controls after 14 days in MC (H4534). **(Q-R)** RT-qPCR-assessed mRNA levels of MECOM and IL12Rβ2 in HL-60 cells virally expressing shRNA targeting MECOM **(Q)** or IL12Rβ2 **(R)**. **(S)** Number of colonies formed by THP-1 cells expressing shRNA targeting MECOM or shIL12Rβ2 compared to the Scramble controls after 14 days in MC (H4534). **(T-U)** RT-qPCR-assessed mRNA levels of MECOM and IL12Rβ2 in THP-1 cells virally expressing shRNA targeting MECOM **(T)** or IL12Rβ2 **(U)**. All experiments (except targeting of INPP4B) have been performed in 2 independent series using 2 shRNA per target. Statistically significant differences to the Scramble controls were calculated by using 1-way ANOVA **(D, I, J, P & S)** to test for significance and data are represented as mean±SEM (*: $p<0.05$; **: $p<0.01$; ***: $p<0.001$; ****: $p<0.0001$). For bar- and dot-plot figures, source data are provided in the Source Data file.

Description of additional supplementary data files:

Suppl. data file 1:
scRNAseq: Differential expression analysis of all cell clusters in TPO vs PBS-exposed cells at day 2.

Suppl. data file 2:
DEG counts in TPO vs PBS comparison (FDR <= 0.05).

Suppl. data file 3:
Selected affected pathways in TPO vs. PBS-exposed cells (related to Fig.3G&K). Two-sided t-test of the camera function from the limma R package was used for statistics analysis.

Suppl. data file 4:
scRNAseq: Differential expression analysis of all cell clusters in TPO vs PBS exposed cells at day 5.

Suppl. data file 5:
Differentially expressed genes in GFP^+ KME AML cells emerging from TPO vs. PBS exposed HSC. Quasi-likelihood F-tests from EdgeR package was used for the comparison.

Suppl. data file 6:
Differentially expressed genes in bulk KME AML cells emerging from TPO vs. PBS exposed HSC. Quasi-likelihood F-tests from EdgeR package was used for the comparison.

Suppl. data file 7:
Differentially expressed genes in GFP^- KME AML cells emerging from TPO vs. PBS exposed HSC. Quasi-likelihood F-tests from EdgeR package was used for the comparison.

Suppl. data file 8:
Statistics of human AML patients analyzed from TARGET, St.JUDE, BEAT and LEUCEGENE databases.

Suppl. data file 9:
Common differentially up-regulated genes in high vs. low Mecom/MECOM mouse KME AML cells and human AML from 4 public databases (FDR <= 0.05).

Suppl. data file 10:
Common differentially down-regulated genes in high vs. low Mecom/MECOM mouse KME AML cells and human AML from 4 public databases (FDR <= 0.05).

Suppl. data file 11:
List of primers used for genotyping KME mice.

Suppl. data file 12:
List of antibodies used for flow cytometry or FACS analysis.

Suppl. data file 13:
List of antibodies used for cell cycle analysis of HSPC by flow cytometry.

Suppl. data file 14:
List of primers used for RT-qPCR analysis.

Suppl. data file 15:
List of antibodies used for Western Blotting.

Suppl. data file 16:
List of antibodies used for single cell RNA sequencing.
