# Supplementary material for: Thrombopoietin increases susceptibility for EVI1 + KMT2A-MLLT3-driven AML expressing stem cell genes linked to poor outcome
Source: Nat Commun. 2025 Dec 19;17:892. doi: 10.1038/s41467-025-67611-w (PMC12830621; doi:10.1038/s41467-025-67611-w)
Supplement: Supplementary file 2 — Description of Additional Supplementary Files [file 41467_2025_67611_MOESM2_ESM.pdf]

Title: Suppl. data 1
Description: scRNAseq: Differential expression analysis of all cell clusters in TPO vs PBS-exposed cells at day 2.

Title: Suppl. data 2
Description: DEG counts in TPO vs PBS comparison (FDR <= 0.05).

Title: Suppl. data 3
Description: Selected affected pathways in TPO vs. PBS-exposed cells (related to Fig.3G&K). Two-sided t-test of the camera function from the limma R package was used for statistics analysis.

Title: Suppl. data 4
Description: scRNAseq: Differential expression analysis of all cell clusters in TPO vs PBS exposed cells at day 5.

Title: Suppl. data 5
Description: Differentially expressed genes in GFP^+ KME AML cells emerging from TPO vs. PBS exposed HSC. Quasi-likelihood F-tests from EdgeR package was used for the comparison.

Title: Suppl. data 6
Description: Differentially expressed genes in bulk KME AML cells emerging from TPO vs. PBS exposed HSC. Quasi-likelihood F-tests from EdgeR package was used for the comparison.

Title: Suppl. data 7
Description: Differentially expressed genes in GFP^- KME AML cells emerging from TPO vs. PBS exposed HSC. Quasi-likelihood F-tests from EdgeR package was used for the comparison.

Title: Suppl. data 8
Description: Statistics of human AML patients analyzed from TARGET, St.JUDE, BEAT and LEUCEGENE databases.

Title: Suppl. data 9
Description: Common differentially up-regulated genes in high vs. low Mecom/MECOM mouse KME AML cells and human AML from 4 public databases (FDR <= 0.05).

Title: Suppl. Data 10
Description: Common differentially down-regulated genes in high vs. low Mecom/MECOM mouse KME AML cells and human AML from 4 public databases (FDR <= 0.05).

Title: Suppl. Data 11
Description: List of primers used for genotyping KME mice.

Title: Suppl. data 12
List of antibodies used for flow cytometry or FACS analysis.

Title: Suppl. data 13
Description: List of antibodies used for cell cycle analysis of HSPC by flow cytometry.

Title: Suppl. data 14
Description: List of primers used for RT-qPCR analysis.

Title: Suppl. data file 15:
Description: List of antibodies used for Western Blotting.

Title: Suppl. data file 16:
Description: List of antibodies used for single cell RNA sequencing.
