# Supplementary material for: Thrombopoietin increases susceptibility for EVI1 + KMT2A-MLLT3-driven AML expressing stem cell genes linked to poor outcome
Source: Nat Commun. 2025 Dec 19;17:892. doi: 10.1038/s41467-025-67611-w (PMC12830621; doi:10.1038/s41467-025-67611-w)
Supplement: Supplementary file 19 — Reporting Summary [file 41467_2025_67611_MOESM19_ESM.pdf]

nature portfolio

Corresponding author(s): Prof. Juerg Schwaller

Last updated by author(s): Apr 16, 2024

Reporting Summary

Nature Portfolio wishes to improve the reproducibility of the work that we publish. This form provides structure for consistency and transparency in reporting. For further information on Nature Portfolio policies, see our Editorial Policies and the Editorial Policy Checklist.

Statistics

For all statistical analyses, confirm that the following items are present in the figure legend, table legend, main text, or Methods section.

n/a	Confirmed	
☐	☒	The exact sample size (*n*) for each experimental group/condition, given as a discrete number and unit of measurement
☐	☒	A statement on whether measurements were taken from distinct samples or whether the same sample was measured repeatedly
☐	☒	The statistical test(s) used AND whether they are one- or two-sided<br>*Only common tests should be described solely by name; describe more complex techniques in the Methods section.*
☒	☐	A description of all covariates tested
☐	☒	A description of any assumptions or corrections, such as tests of normality and adjustment for multiple comparisons
☐	☒	A full description of the statistical parameters including central tendency (e.g. means) or other basic estimates (e.g. regression coefficient) AND variation (e.g. standard deviation) or associated estimates of uncertainty (e.g. confidence intervals)
☐	☒	For null hypothesis testing, the test statistic (e.g. *F*, *t*, *r*) with confidence intervals, effect sizes, degrees of freedom and *P* value noted<br>*Give P values as exact values whenever suitable.*
☒	☐	For Bayesian analysis, information on the choice of priors and Markov chain Monte Carlo settings
☒	☐	For hierarchical and complex designs, identification of the appropriate level for tests and full reporting of outcomes
☐	☒	Estimates of effect sizes (e.g. Cohen's *d*, Pearson's *r*), indicating how they were calculated

Our web collection on statistics for biologists contains articles on many of the points above.

Software and code

Policy information about availability of computer code

Data collection

- RNA-seq was performed on a Illumina NovaSeq 6000 instrument, samples were sequenced with paired-end reads of 51 bases using the TruSeq Stranded mRNA Illumina HT kit. Primary data analysis was performed with STAR (version2.7.9) and SAMtools (version1.15).
For single-cell RNA sequencing; capture, cDNA and library preparation were performed with a 10x Genomics Single Cell 3' v3 Reagent Kit. BioLegend TotalSeq-B antibodies were used to produce hashtag oligos (HTO) and antibody-derived tag (ADT) libraries. Sequencing was performed on three flow-cells of GFB Novaseq 6000 instrument resulting in 90/28nt- and 101/28nt-long paired-end reads. Quality was assessed using FastQC tool (version0.11.5) and reads were aligned with STARsolo (version2.7.9a)
Flow cytometry data were collected using either FACS Diva or LSR II Fortessa (BD, Biosciences) and BD Cytoflex.
- Tissue sections were imaged on a Nikon TI microscope (Nikon, Tokyo, Japan).
- 3D imaging of BM were adapted from previously published protocols. In short, femurs were isolated, cleaned and immersed in PBS/2% paraformaldehyde followed by a dehydration step in 30% sucrose. Femurs were then embedded in cryopreserving medium. Specimens were iteratively sectioned using a CM3050 S cryostat (Leica Biosystems) until the BM cavity was fully exposed. Once BM slices were generated, the remaining OCT medium was removed by incubation and washing of the bone slices in PBS and samples were additionally fixed in 2% PFA/PBS. Confocal microscopy was performed with 10× (HCX PL FLUOTAR), 20× (HC PL APO CS2) and 63x (HCX PL APO CS2) on an SP5 Leica confocal microscope.

Data analysis

- Statistical analysis was performed using Prism software (GraphPad, version 10).
- Flow Cytometry analysis was performed using Flow Jo-Software (Version 10.8).
- Microscopy images were analyzed using Fiji Image J software (version 2.3.0/1.53q).
- 3D whole-mount images were analyzed using the commercially available software Imaris v9 (Bitplane AG, Oxford Instruments, Abigdon-on-Thames, UK).

- RNA-seq analysis was done using R (version4.3.1). featureCount software of Subread (version2.0.1) to count reads, which were then normalized in log Count per Million (CPM) and used for the gene expression plots as well as principal analysis complement (PCA). Differential gene analysis was performed with the EdgeR (version3.36) bioconductor package.

Single-cell RNA-seq data analysis was performed using R (version4.2.0) and mostly following the steps of the workflow presented at https://bioconductor.org/books/3.15/OSCA/. Empty droplets were removed with the emptyDrops function from the DropletUtils package (version1.16.0). The hashedDrops function from the same package was used to demultiplex cell barcodes into their samples. Cells were preclustered using the quickCluster function from the scran20 package (version1.24.0). Sample-specific effects were removed using the fastMNN function from the batchelor package (version1.12.1). Clustering was performed using FindNeighbors and FindClusters functions from the Seurat package (version4.1.1). Doublets were identified and removed using scDblFinder from the scDblFinder package (version1.10.0). Cell cycle phases were assigned to cells using the cyclone function from the scran package (version1.24.0). The dataset was also subjected to cell-type annotations using the package SingleR (version1.10.0). The runTSNE function from the Scater package (version1.24.0) was used to produce T-SNE coordinates and plots. Pseudo-bulk differential expression analysis was performed using the edgeR package (version3.34.1) and gene set enrichment analysis was performed using the camera function from the limma (version3.52.2) package.

Complete blood count were measured on an ADVIA 2120i Hematology System (Siemens Healthineers, Erlangen, Germany).

For manuscripts utilizing custom algorithms or software that are central to the research but not yet described in published literature, software must be made available to editors and reviewers. We strongly encourage code deposition in a community repository (e.g. GitHub). See the Nature Portfolio guidelines for submitting code & software for further information.

Data

Policy information about availability of data

All manuscripts must include a data availability statement. This statement should provide the following information, where applicable:

- Accession codes, unique identifiers, or web links for publicly available datasets
- A description of any restrictions on data availability
- For clinical datasets or third party data, please ensure that the statement adheres to our policy

All sequencing data generated for this publication are in the GEO under accession numbers GSE261134 and GSE261266.
All other materials will be available on request to the corresponding author.

Research involving human participants, their data, or biological material

Policy information about studies with human participants or human data. See also policy information about sex, gender (identity/presentation), and sexual orientation and race, ethnicity and racism.

Reporting on sex and gender	*Use the terms sex (biological attribute) and gender (shaped by social and cultural circumstances) carefully in order to avoid confusing both terms. Indicate if findings apply to only one sex or gender; describe whether sex and gender were considered in study design; whether sex and/or gender was determined based on self-reporting or assigned and methods used.* *Provide in the source data disaggregated sex and gender data, where this information has been collected, and if consent has been obtained for sharing of individual-level data; provide overall numbers in this Reporting Summary. Please state if this information has not been collected.* *Report sex- and gender-based analyses where performed, justify reasons for lack of sex- and gender-based analysis.*
Reporting on race, ethnicity, or other socially relevant groupings	*Please specify the socially constructed or socially relevant categorization variable(s) used in your manuscript and explain why they were used. Please note that such variables should not be used as proxies for other socially constructed/relevant variables (for example, race or ethnicity should not be used as a proxy for socioeconomic status).* *Provide clear definitions of the relevant terms used, how they were provided (by the participants/respondents, the researchers, or third parties), and the method(s) used to classify people into the different categories (e.g. self-report, census or administrative data, social media data, etc.)* *Please provide details about how you controlled for confounding variables in your analyses.*
Population characteristics	*Describe the covariate-relevant population characteristics of the human research participants (e.g. age, genotypic information, past and current diagnosis and treatment categories). If you filled out the behavioural & social sciences study design questions and have nothing to add here, write "See above."*
Recruitment	*Describe how participants were recruited. Outline any potential self-selection bias or other biases that may be present and how these are likely to impact results.*
Ethics oversight	*Identify the organization(s) that approved the study protocol.*

Note that full information on the approval of the study protocol must also be provided in the manuscript.

Field-specific reporting

Please select the one below that is the best fit for your research. If you are not sure, read the appropriate sections before making your selection.

☒ Life sciences ☐ Behavioural & social sciences ☐ Ecological, evolutionary & environmental sciences

For a reference copy of the document with all sections, see nature.com/documents/nr-reporting-summary-flat.pdf

Life sciences study design

All studies must disclose on these points even when the disclosure is negative.

Sample size	The number of animals per experiment as well as the number of biological replicates for each experiment is given in the corresponding figure legends.<br>Good laboratory practice indicates the performance of 3 independent series for in vivo experiments. However, based on the input from Frommlet & Heinze (Lab Anim, 55:65-75, 2021), indicating that 2 out of 3 experiments must be significant, we carried only 2 in vivo experiments when the results reached significance.
Data exclusions	For single-cell RNA-seq analysis, droplets for which the de-multiplexing failed were removed. Initial analysis defined 22 clusters, however clusters "17" to "19" were removed as they could not be investigated for differential expression for lack of sufficient cell numbers.<br>Based on the PCA result during the data exploration, 2 samples (numbers 624 and 35088) were excluded from the RNAseq dataset due to a bad clustering with the observed groups with the other samples.
Replication	Every in vivo experiment was replicated twice. In vitro experiments were performed three times independently
Randomization	Recipient mice were randomly selected for transplantation experiments. For donor mice, groups are defined by genotype so there is no need for randomization.
Blinding	Not relevant for this study

Reporting for specific materials, systems and methods

We require information from authors about some types of materials, experimental systems and methods used in many studies. Here, indicate whether each material, system or method listed is relevant to your study. If you are not sure if a list item applies to your research, read the appropriate section before selecting a response.

Materials & experimental systems

n/a	Involved in the study	
☐	☒	Antibodies
☐	☒	Eukaryotic cell lines
☒	☐	Palaeontology and archaeology
☐	☒	Animals and other organisms
☒	☐	Clinical data
☒	☐	Dual use research of concern
☒	☐	Plants

Methods

n/a	Involved in the study	
☒	☐	ChIP-seq
☐	☒	Flow cytometry
☒	☐	MRI-based neuroimaging

Antibodies

Antibodies used

All antibodies used for FACS and cell cycle analysis of HSPC and the relevant information (dilution, clone, company) are given here and in the Supplementary Tables 12 and 13:

Kit-BV711, BD Biosciences, BD, NJ, USA, Ref. 563160, Clone 2B8, Dilution 1/100
Sca-1-PE-Cy7, BioLegend, San Diego, CA, USA, Ref. 122514, Clone E13-1617, Dilution 1/100
Sca-1-APC-Fire750, BioLegend, San Diego, CA, USA, Ref. 122525, Clone E13-1617, Dilution 1/100
Flt-3 (CD135)-BV421, BioLegend, San Diego, CA, USA, Ref. 135314, Clone A2F10, Dilution 1/50
CD34-APC, BioLegend, San Diego, CA, USA, Ref. 119310, Clone MEC14.7, Dilution 1/100
CD48-BV605, BioLegend, San Diego, CA, USA, Ref. 103441, Clone HM48.1, Dilution 1/100
Slam1 (CD150), eBiosciences, Thermo Fisher Scientific, Waltham, MA, USA, Ref. 12-1502-80, Clone mShad150, Dilution 1/100
GFP-FITC, Rockland Immunnochemicals, Limerick, PA, USA, Ref. 600-402-215, Dilution 1/200
Ki67-PerCP-Cy5.5, BioLegend, San Diego, CA, USA, Ref. 652424, Clone 16A8, Dilution 1/20-1/100

All primary antibodies used for Western Blot and the relevant information (dilution, clone, company) are given here and in the Supplementary Tables 15:

Primary Antibodies:
HOXA9, Abclonal, Düsseldorf, Germany, Ref. A19257
STAT5, Cell Signaling Technology, Danvers, MA, USA, Ref. 94205
STAT5P, Rockland Immunochemicals, Limerick, PA, USA, Ref. 200-301-A45
GAPDH, Cell Signaling Technology, 5174

Secondary Antibodies:
Mouse, Sigma-Aldrich, Merck, Darmstadt, Germany, Ref. NA931VS

Human; Sigma-Aldrich, Merck, Darmstadt, Germany, Ref. NA934VS

All antibodies used for single cell RNA Sequencing and the relevant information (dilution, clone, company) are given here and in the Supplementary Tables 16:

Antibodies for FACS
Kit-BV711, BD Biosciences, BD, NJ, USA, Ref. 563160, Clone 2B8, Dilution 1/100
Sca-1-PE-Cy7, BioLegend, San Diego, CA, USA, Ref. 122514, Clone E13-1617, Dilution 1/100
Flt-3 (CD135)-BV421, BioLegend, San Diego, CA, USA, Ref. 135314, Clone A2F10, Dilution 1/50

CITE-seq labelling antibodies
CD34, BioLegend, San Diego, CA, USA, Ref. TotalSeqTM-B823, Clone MEC14.7, Dilution 1/100, Barcode sequence AAACTCAGGTCCTTC
CD48, BioLegend, San Diego, CA, USA, Ref. TotalSeqTM-B429, Clone HM48.1, Dilution 1/200, Barcode sequence AGAACCGCCGTAGTT
Slam (CD150), BioLegend, San Diego, CA, USA, Ref. TotalSeqTM-B203, Clone TC15-12F12.2, Dilution 1/200, Barcode sequence CAACGCCTAGAAACC

Hashtag labelling antibodies
CD45/MHCI, BioLegend, San Diego, CA, USA, Ref. TotalSeqTM-B301, Clone "HTO 1", Dilution 1/100, Barcode sequence ACCCACCAGTAAGAC
CD45/MHCI, BioLegend, San Diego, CA, USA, Ref. TotalSeqTM-B302, Clone "HTO 2", Dilution 1/100, Barcode sequence GGTCGAGAGCATTCA
CD45/MHCI, BioLegend, San Diego, CA, USA, Ref. TotalSeqTM-B303, Clone "HTO 3", Dilution 1/100, Barcode sequence CTTGCCGCATGTCAT
CD45/MHCI, BioLegend, San Diego, CA, USA, Ref. TotalSeqTM-B304, Clone "HTO 4", Dilution 1/100, Barcode sequence AAAGCATTCTTCACG

Validation

All the antibodies used for flow cytometry analysis are commercially available and are suitable for each species, except the anti-Mpl antibody. Suppliers validated their antibodies by immunofluorescence staining of mouse cells followed by flow cytometry analysis and comparison with isotype control. The Mpl Rabbit polyclonal antibody was made in house and would be available on request from Prof. Wei Tong (Division of Hematology, Children's Hospital of Philadelphia, Philadelphia, USA).
Western blot antibodies were bought commercially and validated by the companies as well as antibodies used for single cell RNA sequencing.

Eukaryotic cell lines

Policy information about cell lines and Sex and Gender in Research

Cell line source(s)	Lenti-Xtm Hek293T-LX obtained from Takara (cat. # 632180)<br>MOLM-13 obtained from DSMZ (cat. # ACC 554)<br>OCI-AML4 obtained from DSMZ (cat. # ACC 729)<br>HL-60 obtained from DSMZ (cat. # ACC 3)<br>THP-1 obtained from DSMZ (cat # ACC 16)
Authentication	Cell line were not authenticated.
Mycoplasma contamination	The cells were tested when needed for Mycoplasma.
Commonly misidentified lines (See ICLAC register)	No commonly misidentified cell lines were used in the study.

Animals and other research organisms

Policy information about studies involving animals; ARRIVE guidelines recommended for reporting animal research, and Sex and Gender in Research

Laboratory animals	B6.SJL-Ptprc<a> Pepc<b>/BoyJ from Jackson Laboratory.<br>B6.129P-Tg(KMT2A-MLLT3)1Apet generated in house. More information regarding this line can be found at 10.1016/j.ccell.2016.05.011 or by contacting the corresponding author.<br>B6;129P-Mecom<tm2Miku> obtained from Prof. Mineo Kurokawa (Department of Haematology and Oncology, Graduate School of Medicine, University of Tokyo, Tokyo, Japan). More information regarding this line can be found at 10.1084/jem.20110447 or by contacting the Prof. Mineo Kurokawa.<br>Mice were kept under specific pathogen-free conditions at the animal facility of the Department of Biomedicine (University of Basel, Basel, Switzerland) with free access to food and water in accordance to Swiss Federal Regulations.
Wild animals	This study did not involve the use of wild animals.
Reporting on sex	Only female mice were used in this study. Due to the fact that the iKMT2A-MLLT3 transgene is integrated into the Hprt gene locus on the X chromosome, we only use female mice for these experiments (as described in the original report that characterized this transgenic line: 10.1016/j.ccell.2016.05.011)

Field-collected samples	This study did not involve field-collecting samples.
Ethics oversight	All experiments were done in adherence to Swiss laws for animal welfare and approved by the Swiss Cantonal Veterinary Office of Basel-Stadt, Switzerland (License number 2087_31838).

Note that full information on the approval of the study protocol must also be provided in the manuscript.

Plants

Seed stocks	N/A
Novel plant genotypes	N/A
Authentication	N/A

Flow Cytometry

Plots

Confirm that:

- [x] The axis labels state the marker and fluorochrome used (e.g. CD4-FITC).
- [x] The axis scales are clearly visible. Include numbers along axes only for bottom left plot of group (a 'group' is an analysis of identical markers).
- [x] All plots are contour plots with outliers or pseudocolor plots.
- [x] A numerical value for number of cells or percentage (with statistics) is provided.

Methodology

Sample preparation	A detailed protocol for cell preparation for FACS analysis and sorting is described in the materials and method sections. In summary, total mouse bone marrow were treated with red blood lysis and then either analyzed directly or depleted for lineage-marker expressing cells prior to analysis.<br>Cells were always washed twice with PBS (2X) prior to 1h incubation for staining in buffer (5mM EDTA, 0.5% BSA, PBS) at 4°C. Before analysis, the cells were washed with buffer (5mM EDTA, 0.5% BSA, PBS) and then resuspended in buffer.
Instrument	Flow cytometry was performed in a LSR II Fortessa (BD, New Jersey, USA).<br>Fluorescence activated cell sorting was performed on a BD FACS Aria III.
Software	FlowJo v10.8 Software (BD Life Sciences, Franklin Lakes, NJ, USA).
Cell population abundance	Post sorting purity was always <95% and was determined (when possible) by reanalysis on LSR II Fortessa machine.
Gating strategy	Gating Strategy as the following: Cells population (FSC-A vs. SSC-A), single cells population (FSC-A vs. FSC-H), and gated according antigen/fluorescence used; according to unstained control and positive and negative beads.<br>For cell cycle analysis, the Fixation and Permeabilization solution kit (Cat. 554714, BD Biosciences, BD) was used.<br>A detailed gating strategy is provided in Supplementary Figure 6.

- [x] Tick this box to confirm that a figure exemplifying the gating strategy is provided in the Supplementary Information.
